# The Service of Dedication at St. Paul's

**Published:** 1917-04-28

**Authors:** 


### THE SERVICE OF DEDICATION AT ST. PAUL'S.

### A Momentous Event in the World's History.

The solemn service to Almighty God on Friday, April 20, 1917, on the occasion of the entry of the United States of America into the Great War of Freedom, will be memorable for all time. Speaking for America, Dr. Charles Henry Brent, Bishop of the Philippine Islands, the preacher, declared "As Christians it would be wicked to ally ourselves with any purpose that we could not take to God's House and ask for His blessing thereon. To-day we have this great confidence—not that our cause is God's in the sense of our winning Him to our position, but in the sense that God has won us to His position." "England, thank God," he declared, "is the home of democracy, and England's children come back to-day and pour all their experience, the experience of a century and a half of independent life, with gratitude at the feet of their mother. Our sympathy for the sufferers has risen into a participation in their sufferings, and to-day we stand side by side with our fellows as common soldiers in the common fight."

### The "Wild Beast" of War.

"Our quarrel with Germany is not a misunderstanding; it is an understanding. We know the principle which that nation has espoused as its guiding star, and that principle is one which contradicts the principle by which men live. America has found her soul. Our immediate purpose is to seal here our pledge to sacrifice our lives, our fortunes, and all we possess to the cause of God and humanity. At last the day has come when America is privileged to spend her blood and might for the principles that gave her birth, for the happiness and peace which she has treasured. God helping her, she can do no other. America with the Allies is fighting for peace. She thinks so much of peace that she is ready to pay the cost of war. Our war to-day is that we may destroy war. War is a wild beast; it cannot be tamed by conferences and conventions, the one thing to do with war is to hunt it to its death—and, please God, in this war we shall achieve our purpose. The soul of democracy is not religion, but organised religion. The day is past for individualistic attempts to redeem mankind by visions that are not tuned to the infinite, the eternal, and the universal. It is becoming increasingly clear that the question of world peace and of Christian reunion go together, for only the visible unity of the Church of Christ will be competent to remove the obstacles in the way of the establishment of any kingdom of peace and righteousness and love. It is true the world is craving for the unity that comes from God, that is maintained by the operation of the Spirit of God. That unity is going to come just as fast as we will let God bring it to us. The only obstacle is our stubbornness."

Then the preacher spoke with telling force the truth which has been insisted upon in these columns often. There is, he declared, a "Prussianism in the Churches to-day, and the watchword of the Churches must be Unity. Either the Churches must justify their claim to be the favoured or exclusive residence of God by exhibiting in their works a holiness or a superiority nowhere apparent, or else let us admit the favour of God towards other Churches of lesser pretensions. A large part of the public has already served notice on the Churches that unless we observe the elementary principles of peaceableness and fairness and fellowship they will get on without us. God defend us from the day when the sheep of Christ's flock turn upon the shepherds because of the shepherds' littleness and inability to be true leaders." "But I see a vision," he proceeded, "I see a great movement, a movement not of man but of God, coming sweeping through this world of ours and gathering into its embrace all true-hearted men. I see a united Church—a Church worthy of the residence of Jesus Christ among men—a Church which will bring holiness and power to all the people of God. That is the end of the vision, and that is the supreme end to which we must commit ourselves to-day as Christian men."

### The "Star Spangled Banner."

Dr. Brent expressed the will and wish of the great congregation assembled in St. Paul's at this service of dedication. There were present the King and Queen, Queen Alexandra, Princess Mary, Princess Victoria, and other princes and princesses, who were attended by British statesmen of eminence, including Lord Rosebery, Mr. Bonar Law, Mr. Asquith, the Prime Ministers of Oversea British Dominions, great British sailors and soldiers, including Admiral Sir John Jellicoe, and Admiral Sims of the United States Navy, Viscount French and Sir William Robertson, and eminent lawyers including the Lord Chancellor and Lord Chief Justice. The King and Queen were met at the west door by the United States Ambassador, other ambassadors present being those of France, Italy, Japan, the representatives of Russia, Belgium, Rumania, and other Allied countries. The clergy present included the Archbishop of Canterbury, the Bishop of London, the Bishop of the Philippine Islands, and other bishops. The service in every particular was worthy of the occasion, and demonstrated once again the admirable administration of those responsible for St. Paul's Cathedral in every detail.

Congregations differ as the stars in the heavens. Reverence at the Dedicatory Service was the paramount note which made itself felt, and produced a harmony in the singing by the congregation that went straight to the heart, and will not be forgotten by those present. The hymns chosen were in tune with the solemnity of the occasion. The whole attention of everybody present was concentrated on the hymn "O God, our Help in Ages past," and memories of the war and what it has brought to so many families entered into the second hymn, "Through the Night of Doubt and Sorrow." The music selected and played by the Welsh Guards' band was very beautiful and appropriate. This band played the "Battle Hymn of the Republic" and "The Star-Spangled Banner," which with the National Anthem concluded the service, with such spirit that the whole congregation were carried away by them, and the strength, depth, and appealing force of the American hymns were expressed with an emotion and verve always to be remembered. We hope America's joining hands with the Allies may revive and clinch the interest of every section of the American people in these two national hymns, so that every one of the rising generation may come to know them well and love them with a depth of apprehension that will give added national strength to our brothers across the sea. The uniting of the Anglo-Saxon race should give all Americans a keen appreciation of their National Anthems and bring them into a prominence from which we venture to hope they will never again be withdrawn. The Welsh Guards' band won for itself a reputation for excellence which is likely to grow and endure.

It was a Great Service of Dedication, a Solemn Service to Almighty God on the occasion of the uniting of the English-speaking Nations, and the entry of the United States of America into the Great War for the freedom of humanity. The music was fitting to the occasion. It was solemn, heart-stirring, uplifting, and beautiful, and its effect upon those present must endure and help them often as the days pass.

